# Evaluating Vaccination Coverage and Willingness to Participate in Immunization Programs Among People Living in Prison: A Cross-Sectional Survey in Italy

**DOI:** 10.3390/vaccines14070600

**Published:** 2026-07-07

**Authors:** Giovanna Paduano, Vincenza Sansone, Vincenzo Aiello, Gabriella Di Giuseppe, Maria Pavia

**Affiliations:** 1 Department of Experimental Medicine, University of Campania, “Luigi Vanvitelli”, Via Luciano Armanni 5, 80138 Naples, Italy; giovanna.paduano@unicampania.it (G.P.); vincenza.sansone@unicampania.it (V.S.); vincenzo.aiello@unicampania.it (V.A.); gabriella.digiuseppe@unicampania.it (G.D.G.); 2 Distretto Socio-Sanitario 34, Azienda Sanitaria Locale Napoli 3 Sud, Piazzale Gradoni 26, 80055 Naples, Italy

**Keywords:** vaccination uptake, vaccine willingness, prison population, cancer screening, health information

## Abstract

Background/Objectives: People living in prison are at increased risk of communicable diseases due to overcrowding and suboptimal living conditions. This study aimed to estimate the uptake of recommended vaccinations among people living in prison, investigate their willingness to be vaccinated during detention, and identify factors influencing both uptake and willingness. Methods: This cross-sectional survey was conducted from October 2023 to July 2024 in four penitentiary institutions in Southern Italy. Results: Of the 1137 participants, 13.8% self-reported having received at least one vaccination schedule during their current detention. Those with chronic disease, who had received information about vaccines in prison, and with a longer detention were significantly more likely to have received a vaccination. Conversely, women, those not-at-risk from alcohol consumption, and those having at least one child were significantly less likely to have received at least one vaccination schedule in prison. Less than half of the participants (41.9%) expressed willingness to receive recommended vaccinations if offered in prison. Willingness was significantly higher in subjects of Italian nationality and with a longer detention. Moreover, heterosexuals and those who were married or cohabitant were significantly less willing to receive at least one vaccination schedule in prison. Conclusions: Implementing routine proactive vaccination activities could be effective, and should focus on both standard adult vaccinations and immunizations for individuals with specific risk conditions.

## 1. Introduction

Globally, approximately 11 million people are currently living in prisons and other detention facilities. In Western Europe, the median incarceration rate is 73 per 100,000, and in Italy, by the end of 2024, the incarcerated population had reached 62,153 individuals, significantly exceeding the official regulatory capacity of 51,320 places and resulting in an effective overcrowding rate of 132.6% [1]. Due to high population density and vulnerable conditions, the risk of contracting communicable diseases is elevated for people living in prisons (PLP) [2,3], and the prevalence of infectious diseases in this population is significantly higher than in the general community, which underscores the crucial importance of preventive measures [4,5]. While it is widely acknowledged that vaccination is an effective public health tool to decrease morbidity and mortality, many prevalent communicable diseases in prisons that are vaccine-preventable continue to pose a threat [6]. In several countries, PLP face significant difficulties in accessing health services, and specifically preventive health services. This gap not only puts the health of PLP at risk, but also impacts wider public health, considering the frequent exchange of people between prisons and the community. In Italy, the provision of healthcare services to PLP is under the responsibility of the National Health Service (NHS) and should be the same as for all citizens. According to the National Vaccine Prevention Plan (NVPP), vaccinations for PLP are offered free of charge, voluntarily, and are not mandatory. This principle applies universally to all PLP, including foreigners [7]. Upon admission to penitentiary institutions, a medical examination is legally mandatory to assess the health status and healthcare needs of the incoming PLP. During this entry visit, screening for infectious diseases (such as HIV, hepatitis B, and hepatitis C) is actively offered but strictly requires informed consent, meaning testing is not legally mandatory. The evaluation of immune status before vaccination is highly recommended for this specific target population only for hepatitis B. Moreover, this initial evaluation in prison is often useful only to start a care plan, but not a prevention program.

There is evidence that PLP are often not adequately vaccinated, particularly for critical vaccines such as hepatitis B, influenza, and measles, mumps, and rubella (MMR) [4,6]. Recent research has highlighted that while vaccines are available in various European prison settings, there are significant differences based on the country and the type of vaccine [5]. Despite these findings, implementing interventions to increase vaccination uptake among both PLP and prison staff remains a global challenge [8]. Previous research conducted in European and international prison settings has highlighted considerable heterogeneity in vaccine availability, delivery strategies, and coverage rates [5,9]. Studies have reported that although vaccines are theoretically available, their actual implementation is often inconsistent, with significant differences across institutions and regions [5,10]. This variability reflects differences in national policies, healthcare organizations, and resource allocation within correctional systems.

Despite the growing body of literature on infectious diseases in prison settings, important gaps remain regarding vaccination practices and attitudes among PLP, particularly in the Italian context. Limited data are available on both the actual provision of recommended vaccines and individuals’ willingness to be vaccinated during incarceration. Moreover, the factors influencing vaccine acceptance in this population are still not fully understood. Addressing these gaps is essential to inform targeted interventions and improve the effectiveness of vaccination strategies in correctional facilities. Moreover, epidemiological data on immunization programs within prison facilities are fragmented; indeed, the vast majority of worldwide previous studies have focused on single, specific pathogens [10,11,12,13,14]. While these studies offer valuable insights into control of single specific diseases, they fail to capture the broader, systemic determinants of immunization behaviors among PLP. Consequently, there is a distinct gap in the literature regarding how this vulnerable population interacts with the entire panel of routinely recommended adult vaccinations.

Therefore, it seemed interesting to conduct a survey to achieve the following primary objectives: to estimate the provision of recommended vaccinations among PLP, to investigate the willingness to be vaccinated while incarcerated, and to identify potential influencing factors, hypothesizing that higher education and health factors (such as having a chronic disease) would positively influence both willingness and uptake of vaccinations, and that institutional exposure would act as a powerful driver to confirm vaccination adherence, whereas high-risk behaviors (e.g., substance abuse) would negatively correlate with these outcomes.

## 2. Materials and Methods

### 2.1. Study Design

This cross-sectional survey is part of a larger project developed by the University of Campania “Luigi Vanvitelli” and the Joint Operational Unit for “Health Protection at Prison Institutions”, dedicated to examining various health-related conditions affecting PLP [15,16,17,18]. Participants were recruited from October 2023 to July 2024 from four correctional facilities located in the Campania region, Southern Italy. The study population was defined according to prespecified inclusion and exclusion criteria. Inclusion criteria comprised all PLP present in the selected institutions at the beginning of the study, as well as all consecutive admissions recorded during the observation period. Eligibility for each vaccination was ascertained according to age, presence of chronic disease(s), and vulnerability criteria as defined by the most recent NVPP [7]. Conversely, exclusion criteria included individuals held in special units or those with significant cognitive impairments.

### 2.2. Data Collection

Once the prison director’s approval was secured, researchers met PLP to illustrate the study’s objectives and invite them to take part in a face-to-face interview. Participants were clearly informed that involvement was entirely voluntary, data would be handled anonymously, and that declining or discontinuing participation would not affect their access to healthcare services nor their prison conditions. No incentives or rewards were offered. Each participant received a standardized oral explanation of the interview objectives and was invited to provide verbal consent to participate. After obtaining verbal consent in the presence of a correctional officer, who served as a witness, a trained researcher conducted a face-to-face interview with each participant in a semi-private area within the prison, with the interviewer and participant seated in front of each other at a table. The use of verbal consent was considered the most appropriate and effective approach to ensure understanding and to promote participation, as substantial evidence supports alternatives to written consent in detained populations to enhance comprehension and foster trust [19,20]. Furthermore, reluctance to sign documents has been recognized as a barrier to participation in prison-based research [19], and factors such as limited literacy, language difficulties, and reduced comprehension skills in this population may lead to misunderstanding or mistrust when written consent forms are used [21]. The data collection sessions lasted about 20 min. Although a correctional officer was stationed nearby for safety purposes, they remained far enough away to protect the secrecy of the conversation and the participant’s privacy.

### 2.3. Questionnaire Design

The questionnaire was designed on the basis of validated items used by the research team in previous surveys on vaccinations [7,15,16,22]. The sections of the questionnaire that were used for this study were the following: (1) socio-demographic, detention, anamnestic, and lifestyle characteristics; (2) information regarding the recommended vaccination in prison and vaccination uptake in eligible subjects in and outside prison and the reasons influencing their decisions—in particular, catch-up and booster vaccinations (Diphtheria/tetanus/pertussis [dTpa] and Measles/mumps/rubella/varicella [MMRV]), cancers-preventing vaccines (hepatitis B and papillomavirus), vaccinations for individuals with specific conditions (meningococcal ACW135Y [MenACWY], meningococcal B [MenB], hepatitis A, pneumococcal, influenza, and herpes zoster)—were investigated; (3) willingness to be vaccinated, if offered in prison, was also investigated with the reasons influencing their decisions; (4) participation in cervical, breast, and colorectal cancer screening programs among eligible participants was also assessed. Alcohol-related disorders were explored using the Alcohol Use Disorders Identification Test-Concise (AUDIT-C) to classify PLP into the three categories of consumer not at risk, harmful consumption, or alcohol dependence [23]. The questionnaire was translated into English and was available for interview submission to participants who did not speak Italian and understood the English language. A pilot study on 50 PLP was performed to test the clarity, feasibility, and reliability of the questionnaire, and the pilot study results were used in the final sample, since no modifications needed to be made to the survey instrument.

### 2.4. Statistical Analysis

All analyses were performed with Stata software, version 17 [24]. First, descriptive statistics (frequencies, means, and standard deviations) were calculated. Then, two multilevel mixed-effects logistic regression models were designed for the following outcomes: having received at least one vaccination schedule in prison (no = 0; yes = 1) (Model 1) and willingness to receive at least one vaccination schedule in prison (no = 0; yes = 1) (Model 2). With the aim of accounting for the multilevel dataset structure (PLP and related immunization pattern are “nested” within prison facilities), the variable “prison facility” was introduced in the models as a random factor. The models were developed according to Hosmer and Lemeshow’s strategy [25], including bivariate analysis, using the appropriate test statistic (chi-square test and Fisher’s exact test), and the choice of the best fit to include determinants in the model (continuous, dichotomous, or categorical), or the better model fit, to provide a more accurate and powerful estimation of the overall model; then, variables with a *p* value ≤ 0.25 at the bivariate test or that were judged to potentially influence the investigated outcomes were included in the model. Specifically, socio-demographic characteristics, such as higher education levels, employment before detention, and chronic disease, were hypothesized to significantly increase both vaccination uptake and willingness due to greater health literacy and higher perceived vulnerability. Detention-related variables, such as a longer detention and having received formal health or vaccine-related information within the prison, would positively influence vaccine uptake. Conversely, lifestyle factors, such as alcohol or drug abuse, would represent potential barriers, correlating with lower adherence to preventive interventions and reduced willingness to participate in institutional vaccination programs. The independent variables included in the different final models and the related reasons for inclusion are shown in the Supplementary Material S1. Adjusted odds ratios (ORs) and 95% confidence intervals (CIs) were calculated. All reported *p* values are two-tailed, and a value ≤ 0.05 is considered statistically significant. Intraclass correlation coefficients (ICCs) were calculated to estimate the potential clustering effect. For both models, multicollinearity among the fixed-effects predictors was checked through the variance inflation factor (VIF). Missing data on the variables included in the models were handled using listwise deletion; consequently, only participants with complete information for all variables in the final models were included in the analysis.

### 2.5. Ethics

This study was approved by the Ethics Committee Campania 2 (protocol code: N.0029553/12 October 2023).

## 3. Results

Out of 1391 PLP who were contacted to participate in the study, a total of 1137 were surveyed, with a response rate of 81.7%. Specifically, recruitment and response rates in the four prison institutions were as follows: Prison 1: 376/424 (88.7%); Prison 2: 188/220 (85.5%); Prison 3: 435/580 (75%); and Prison 4: 138/167 (82.6%). Despite this high response rate, a proportion of PLP declined to participate in the study, and the most frequently reported reasons were lack of trust in the healthcare provided in prison, no interest in the project, and considering medications administered within the facility, including vaccines, to be substandard. The main characteristics of the participants are shown in Table 1 and Table 2. The mean age was 44.5 years (SD ± 12.1, range 18–83), almost all were males (84.9%), a large majority were Italians (91.5%), almost two-thirds (62.1%) were married or cohabitant, 18.7% had no more than a primary school degree or no education, and 22.4% were unemployed before detention. Almost half of the sample (47.6%) had already had other episodes of detention, with a mean time spent in prison of 6 years (SD ± 7.8, range < 1–44). Almost all (98.2%) lived in shared cells, only 30.3% were involved in some working activity in prison, and only 2.8% declared having received information regarding recommended vaccination at their arrival in prison (Table 1). Overall, 28.7% of the PLP reported being affected by at least one chronic disease. Three-quarters (77.3%) were current smokers, and 20.5% were considered at risk of alcohol abuse according to the Audit-C score (Table 2).

While the vast majority (94.4%) reported having undergone dTpa during their life, only 31.3%, 4.8%, and 3.8%, respectively, reported having been vaccinated against hepatitis B, papillomavirus, and MMRV. When asked about vaccinations received in prison, prevalence ranged from 0% for papillomavirus vaccination to 7.5% for hepatitis B, with 0.9% for dTpa and 1.4% for MMRV. Among those eligible, willingness to be vaccinated in prison ranged from 24.4% for papillomavirus to 41.9% for dTpa, with 25.8% for MMRV and 38.2% for hepatitis B (Table 3).

Regarding vaccinations for individuals with specific conditions, one-fourth (26.7%) reported having undergone influenza vaccination during their life, while only 2.9%, 2.8%, 1.9%, 1.3%, and 0.4%, respectively, reported having been vaccinated against hepatitis A, pneumococcal, MenACWY, MenB, and herpes zoster diseases. When asked about vaccinations received in prison, prevalence ranged from 0.4% for herpes-zoster to 19.1% for influenza vaccination, with 0.6% for MenACWY and MenB, 0.8% for pneumococcal, and 2.9% for hepatitis A vaccinations. Among those eligible, willingness to be vaccinated in prison ranged from 31.4% for influenza to 41.5% for MenB, with 33.7% for pneumococcal vaccination, 34.4% for herpes zoster, 38.2% for hepatitis A, and 40.4% for MenACWY vaccination (Table 3).

Overall, 13.8% of the sample declared having received at least one vaccination schedule in prison, and among those who had not undergone at least one vaccination schedule for which they were eligible, only 41.9% expressed willingness to receive at least one in prison.

Among PLP who were vaccinated during their life, the most common reported reason was the mandatory nature of the vaccine, particularly for dTpa (92.8%) and hepatitis B (53.1%). Moreover, a physician’s recommendation was the main reason cited by 50% of those who received the papillomavirus and MenB vaccines, while feeling at risk of infection was the primary reason for uptake of herpes zoster (66.7%) and pneumococcal (42.7%) vaccination. In contrast, the main reasons for not having undergone vaccinations included having already contracted the disease, as reported by 89.5% for MMRV, and the perception that the vaccination was not useful, reported by 64.3% and 43.3% of those who had not undertaken the papillomavirus and the hepatitis A vaccine, respectively. Furthermore, structural and informational barriers were reported by 28.3% who were unaware they could receive the MenB vaccine, and 20.8% who stated they were not offered influenza vaccination while in prison (Supplementary Material S2).

For PLP who were vaccinated during their incarceration, the leading motivators were the direct offer of the vaccine within the facility, particularly for influenza (43.9%), and the recommendation by a physician, which was the sole reason cited for both MenACWY and MenB and the most common reason for MMRV (46.7%). Moreover, mandatory vaccine requirements significantly influenced uptake in prison for hepatitis B (42.7%) and dTpa (28.6%) (Supplementary Material S2).

Among PLP who expressed willingness to be vaccinated in prison, the primary reasons were the perceived reduction of infection risk, reaching 55.6% for papillomavirus and 41.3% for MenB, and the feeling of being at risk of infection, cited by 37.6% of those eligible for the pneumococcal and 35.7% for the influenza vaccine. In contrast, the most frequent reasons for unwillingness were the belief that the vaccination is not useful, peaking at 90% for papillomavirus and 69% for hepatitis A vaccine, as well as a low perception of personal risk, the main deterrent for both the dTpa (43.7%) and hepatitis B (38.6%) vaccines (Supplementary Material S2).

The results of the univariate analysis for the two main outcomes of interest are reported in Table 1 and Table 2, whereas the results of the multivariate analysis about having received and willingness to receive at least one vaccination schedule in prison are reported in Table 4.

Specifically, having received at least one vaccination schedule in prison was significantly more likely in those suffering from at least one chronic disease (OR = 2.15, 95% CI = 1.44–3.22), those who reported having received information regarding the recommended vaccinations in prison (OR = 4.49, 95% CI = 1.65–12.22), and among those with a longer detention (OR = 1.05, 95% CI = 1.03–1.08); moreover, those not-at-risk from alcohol consumption (OR = 0.58, 95% CI = 0.34–0.98), those having at least one child (OR = 0.52, 95% CI = 0.33–0.81), and women (OR = 0.39, 95% CI = 0.19–0.81) were significantly less likely to have received at least one vaccination schedule in prison. The ICC was approximately zero (ICC = 6.93 × 10^−35^; 95% CI: not estimable), indicating negligible between-institution variability (Model 1 in Table 4). When the analysis was restricted to PLP eligible for at least one cancer screening test, it resulted that having participated in at least one organized cancer screening program in prison was significantly associated with having received at least one vaccination schedule in prison (OR = 3.25, 95% CI = 1.52–6.91). Moreover, willingness to receive at least one vaccination schedule in prison was significantly more likely in Italian PLP (OR = 2.05, 95% CI = 1. 09–3.86) and among those with a longer detention (OR = 1.03, 95% CI = 1.01–1.06). Moreover, heterosexuals (OR = 0.06, 95% CI = 0.01–0.56) and married or cohabitant (OR = 0.65, 95% CI = 0.44–0.95) individuals were significantly less willing to receive at least one vaccination schedule in prison. The estimated ICC was 0.059 (95% CI: 0.013–0.232), indicating that approximately 6% of the total variance in willingness to uptake vaccination was attributable to between-institution variability (Model 2 in Table 4).

In both models, no relevant collinearity was found, with VIF ranging from 1.02 to 1.93 (Mean VIF = 1.38) in Model 1, and from 1.02 to 2.06 (Mean VIF = 1.36) in Model 2.

## 4. Discussion

This is one of the few studies that has investigated the comprehensive vaccination needs of a large population of PLP in Italy, as well as their willingness to be vaccinated. The findings of this study may provide valuable insights for the development of strategies aimed at improving immunization coverage in this underserved population. There is a paucity of information on vaccination coverage in PLP; data are sparse, and most studies have analyzed single vaccinations, predominantly COVID-19, hepatitis B, influenza, and more recently papillomavirus [4,5,26]. Moreover, studies involving other vaccinations, such as those against measles, varicella, pneumococcal, etc., mostly described responses to existing outbreaks that occurred in PLP [9]. Conversely, the rationale of this study, given that in Italy provision of healthcare services to PLP is under the responsibility of the Ministry of Health and should be the same as for all citizens, was to assess to what extent healthcare services address and satisfy PLPs’ vaccination needs according to the NVPP on a regular basis. Therefore, the finding that only an irrelevant minority (2.8%) had been informed about recommended vaccinations upon arrival in prison is concerning, but somehow expected; although at the time of incarceration PLP in Italy undergo a medical examination to collect information on health status and healthcare needs, it has been reported that guidelines and policies on prison health do not adequately reflect the relevance of proactive and preventive interventions, such as vaccinations [26]. Accordingly, the provision of vaccinations among eligible PLP during incarceration was reported to be very low for all vaccinations, ranging from 0% for papillomavirus to 19% for seasonal influenza vaccination. The unsatisfactory vaccination uptake in prison is coupled with the low vaccination coverage in the investigated PLP, which is in line with previous studies indicating that incarcerated people are under-immunized at arrival in prison [9]. These results confirm that, in the investigated area, subjects arriving in prison belong to underserved groups, but also that the opportunity to contrast health inequalities by promoting vaccinations in prison is not yet perceived by health authorities involved in the delivery of health services to PLP. Moreover, there seems to be no substantial differences among vaccinations, which are not provided on a regular basis, with influenza and hepatitis B being more frequently offered, and all other investigated vaccinations being only very sporadically delivered to eligible PLP. The analysis of the main reasons reported for accepting or refusing vaccination is very informative, since it supports the relevant role of recommendation by trustworthy sources, such as physicians, regarding the awareness of risk of vaccine preventable diseases (VPD) and of the effectiveness of vaccinations, as well as of the active offer of vaccinations in prison. These findings are in line with previous studies, indicating the role of trusted individuals and the availability of vaccines as factors facilitating vaccine uptake in prison [9,27], coupled with the relative absence of barriers for the delivery of vaccinations when offered by healthcare workers (HCW) and performed on-site [16,27,28]. Moreover, they indicate the need for educational programs aimed at developing awareness of the risks of VPD and regarding the effectiveness of vaccinations, which should involve HCW [16,27] or trained PLP as peer educators [29,30]. This study has also provided insight into the characteristics that influence adherence to vaccinations in PLP, underscoring the importance of having received information on vaccinations in prison as a predictor of uptake; it is of note that vulnerable PLP reporting at least one chronic disease and those who had longer detentions were more likely to have received vaccinations, suggesting the need to provide vaccinations on a regular basis in all eligible PLP, promote comprehensive information and education on the effectiveness of vaccinations, and extend the target not only to vulnerable subjects, but to all eligible PLP. The finding that previous participation in organized cancer screening programs was a predictor of vaccination uptake confirms that adherence to a preventive behavior can trigger a virtuous cycle of adherence to other preventive behaviors, as already reported with PLP in the same area [17,18]. One of the main aims of the study was to assess the potential response of PLP to an active offer of vaccinations in prison, given that no systematic program, except for seasonal influenza vaccination in some institutions, was in place at the time of the survey. The findings of the study have demonstrated that willingness to undergo vaccination is not satisfactory, involving less than half of the eligible PLP for all investigated vaccines, and ranging from 24.4% for papillomavirus to 41.9% for dTpa vaccination. The literature on intention to undergo vaccinations among PLP is scarce and focused on single vaccinations, and shows higher willingness for hepatitis B (82%) [31] and COVID-19 (88.72%) vaccination [32]. This negative attitude has been reported, and vaccine hesitancy in PLP has been associated with concerns about side effects, low levels of perceived risk, and distrust of authorities, the vaccine, or the vaccinator [5], and is consistent with the findings of this study, which showed low perception of risk and distrust in the usefulness of vaccines as reasons for refusing vaccinations if offered in prison. These findings, coupled with those from the multivariate regression analysis, which revealed that willingness to undergo vaccinations in prison was associated with longer periods of detention, indicate the key contribution that contact with HCWin prison has for the development of a positive attitude towards preventive behaviors such as vaccinations. These associations are consistent with what has been reported for other preventive attitudes of PLP, such as organized cancer screening programs [15,17,18].

## 5. Strengths and Limitations

Strengths of this study lie in the large recruited population and in the evaluation of preventive needs for a wide variety of vaccinations, both age-based and risk-based; the study has provided valuable information consistent with the WHO Prison Health Framework for the assessment of prison health system performance [33]. Moreover, the survey allowed the efficient collection of standardized data across a large and traditionally hard-to-reach population, ensuring anonymity and enabling the concurrent evaluation of multiple socio-demographic, clinical, and behavioral variables. However, findings of this survey are prone to potential limitations, which should be addressed for an appropriate interpretation of the accuracy of results. First, since data were collected through a cross-sectional design at a single point in time, the study observes associations but cannot establish causality between potential predictors and outcomes. Second, the data rely on participants accurately recalling and disclosing their own vaccine status, with no objective verification through chart review; therefore, the results are vulnerable to recall and social desirability bias, with PLP reporting socially acceptable attitudes and behaviors while under observation. Indeed, self-reported vaccine history may lead to an over- or underestimation of the true immunization coverage within this setting. Furthermore, since the clinical indications for targeted vaccines were established through self-disclosed chronic diseases and behavioral risk factors rather than a formal medical diagnosis, the eligibility status of certain participants may have been subject to misclassification bias. Third, the findings are drawn from a specific sub-sample of PLP across four prisons in Southern Italy, and it is conceivable to suggest the potential for reduced external validity, meaning the results may not apply to different prison environments across Italy or Europe. Therefore, the generalizability of the results to the wider population of PLP should be made with caution. Consequently, future research should incorporate multi-site sampling across diverse geographic regions to enhance external validity and confirm these associations on a broader scale. Furthermore, some limitations regarding the variables evaluated must be acknowledged. Potentially unmeasured confounding factors were not examined in this survey, such as general vaccine hesitancy and levels of health literacy. Low health literacy is particularly prevalent in correctional facilities and is known to negatively impact the understanding of health benefits, potentially reducing both vaccine uptake and willingness to participate in immunization programs. Future studies should include validated tools to explicitly measure these dimensions. Moreover, despite the overall large sample size, there were restricted sample sizes within specific covariates, such as having received information regarding recommended vaccinations in prison and sexual orientation, which produced unprecise estimates with wide confidence intervals; nevertheless, there was enough power to reveal significant associations with the outcomes, which was our main aim. Finally, although the response rate was high, making the voluntary nature of the sample representative of those specific facilities, the lack of data from PLP who did not participate does not permit the exclusion of selection bias.

Taken all together, the results of the study allowed us to successfully achieve all the objectives of the survey by providing a comprehensive assessment of immunization patterns and attitudes among PLP in Italy. The findings highlight that actual vaccination uptake and willingness during detention remain suboptimal. Crucially, the study demonstrated that a longer detention is a stronger predictor of both willingness to vaccinate and uptake of vaccinations.

## 6. Conclusions

The results of the study have demonstrated that PLP have extensive vaccination needs upon arrival, which are generally neither identified nor managed by prison HCW during the clinical evaluations conducted at entry in prison, and vaccination programs for eligible subjects are not offered on a regular basis. Moreover, the reported findings successfully confirmed most of the primary research hypotheses. Conversely, the influence of high-risk lifestyles as negative predictors was only partially supported, as institutional factors played a far more dominant role. Implementing routine proactive vaccination activities could be effective, and should focus on both standard adult vaccinations and immunizations for individuals with specific risk conditions. Immunization programs should be preceded by educational interventions involving HCW and peer educators, and would be valuable for contrasting disparities of access to preventive services in this vulnerable population.

## Figures and Tables

**Table 1:** Socio-demographic and detention characteristics of PLP participating in the survey and related association with outcomes of interest.

			Having Received at Least One Vaccination Schedule in Prison	Willingness to Receive at Least One Vaccination Schedule in Prison
Characteristics ^#^	Total (N: 1137)		(N: 157; 13.8%) °	(N: 275; 41.9%) ^a^
	N	%	N (%)	N (%)
Socio-demographics				
Gender				
Male	965	84.9	141 (14.6)	243 (42.2)
Female	172	15.1	16 (9.3)	32 (39.5)
			χ^2^ = 3.45, df = 1, *p* = 0.063	χ^2^ = 0.21, df = 1, *p* = 0.647
Age, years	44.5 ± 12.1 (18–83) *			
≤30	155	13.6	43 (27.7)	6 (33.3)
31–40	273	24	30 (11)	68 (42.8)
41–50	353	31.1	22 (6.2)	110 (43.5)
51–60	238	20.9	29 (12.2)	65 (39.4)
>60	118	10.4	33 (28)	26 (41.9)
			χ^2^ = 64.53, df = 4, *p* < 0.001	χ^2^ = 1.27, df = 4, *p* = 0.865
Nationality				
Foreigners	97	8.5	13 (13.4)	20 (31.3)
Italians	1.040	91.5	144 (13.8)	255 (43)
			χ^2^ = 0.01, df = 1, *p* = 0.903	χ^2^ = 3.27, df = 1, *p* = 0.07
Sexual orientation				
Heterosexual	1.119	98.4	154 (13.8)	268 (41.3)
Homosexual/bisexual	18	1.6	3 (16.7)	7 (87.5)
			Fisher’s exact = 0.728	χ^2^ = 6.93, df = 1, *p* = 0.008
Marital status				
Unmarried/widowed/separated/divorced	431	37.9	61 (14.1)	112 (48.3)
Married/cohabitant	706	62.1	96 (13.6)	163 (38.4)
			χ^2^ = 0.07, df = 1, *p* = 0.792	χ^2^ = 6.07, df = 1, *p* = 0.014
Having children	1.9 ± 1.7 (0–13) *			
Yes	848	74.6	100 (11.8)	214 (40.8)
			χ^2^ = 11.39, df = 1, *p* = 0.001	χ^2^ = 1.29, df = 1, *p* = 0.257
Education level				
None	10	0.9	2 (20)	2 (33.3)
Primary school	202	17.8	22 (10.9)	50 (35)
Middle school	720	63.3	106 (14.7)	170 (42)
High school	179	15.7	23 (12.9)	45 (51.1)
University degree	26	2.3	4 (15.4)	8 (53.3)
			Fisher’s exact = 0.569	Fisher’s exact = 0.133
Occupation before detention				
Unemployed	254	22.4	45 (17.7)	55 (38.7)
Employed	881	77.6	112 (12.7)	220 (42.7)
			χ^2^ = 4.14, df = 1, *p* = 0.042	χ^2^ = 0.73, df = 1, *p* = 0.394
Detention related				
Institution				
Prison 1	376	33.1	49 (13)	53 (26.6)
Prison 2	188	16.5	20 (10.6)	40 (44)
Prison 3	435	38.3	74 (17)	162 (52.1)
Prison 4	138	12.1	14 (10.1)	20 (35.7)
			χ^2^ = 7.1, df = 3, *p* = 0.069	χ^2^ = 33.36, df = 3, *p* < 0.001
First detention	595	52.4	76 (12.8)	117 (35.6)
			χ^2^ = 1.15, df = 1, *p* = 0.283	χ^2^ = 10.96, df = 1, *p* = 0.001
Length of detention, years	6 ± 7.8 (<1–44) *			
Working activity in the prison	344	30.3	45 (13.1)	82 (45)
			χ^2^ = 0.21, df = 1, *p* = 0.64	χ^2^ = 1.06, df = 1, *p* = 0.304
Living arrangements				
Individual cell	20	1.8	1 (5)	4 (26.7)
Shared cell	1.117	98.2	156 (14)	271 (42.2)
			Fisher’s exact = 0.342	Fisher’s exact = 0.294
Having received information regarding the recommended vaccination in prison	32	2.8	9 (28.1)	5 (55.6)
			χ^2^ = 5.67, df = 1, *p* = 0.017	χ^2^ = 0.71, df = 1, *p* = 0.402

* Mean ± Standard Deviation (range); ° Eligible participants are all PLP surveyed (Total n: 1137); ^a^ Eligible participants who had not yet received at least one vaccination schedule or recall in the last 10 years for dTpa (Total n: 657). ^#^ Number of each item may not add up to total number of study population due to missing values.

**Table 2:** Anamnestic and lifestyle characteristics of PLP participating in the survey and related association with outcomes of interest.

			Having Received at Least One Vaccination in Prison	Willingness to Receive at Least One Vaccination in Prison
Characteristics ^#^	Total (N: 1137)		(N: 157; 13.8%) °	(N: 275; 41.9%) ^a^
	N	%	N (%)	N (%)
Anamnestic				
At least one chronic disease	326	28.7	68 (20.9)	85 (48)
			χ2 = 19.09, df = 1, *p* < 0.001	χ^2^ = 3.78, df = 1, *p* = 0.052
Having participated in at least one preventing smoking, alcohol or drug program	246	21.6	41 (16.7)	71 (51.5)
			χ^2^ = 2.15, df = 1, *p* = 0.142	χ^2^ = 6.61, df = 1, *p* = 0.01
Having participated in at least one organized cancer screening program ^@^	213	45	36 (16.9)	45 (39.1)
			χ^2^ = 4.31, df = 1, *p* = 0.038	χ^2^ = 0.97, df = 1, *p* = 0.326
Having participated in at least one organized cancer screening program in prison ^@^	81	17.1	21 (25.9)	19 (51.4)
			χ^2^ = 13.45, df = 1, *p* < 0.001	χ^2^ = 1.31, df = 1, *p* = 0.253
Lifestyle				
Smoking status				
Never smokers	197	17.3	24 (12.2)	46 (38.3)
Former smokers	61	5.4	10 (16.4)	15 (46.9)
Current smokers	879	77.3	123 (14)	214 (42.4)
			χ^2^ = 0.81, df = 2, *p* = 0.669	χ^2^ = 0.99, df = 2, *p* = 0.607
Drugs use	577	50.8	83 (14.4)	156 (47.6)
			χ^2^ = 0.32, df = 1, *p* = 0.567	χ^2^ = 8.76, df = 1, *p* = 0.003
Alcohol consumption (Audit-C)				
Never	554	48.8	79 (14.3)	145 (42.4)
Not being at risk of alcohol abuse	348	30.7	34 (9.8)	67 (35.5)
Being at risk of alcohol abuse	232	20.5	43 (18.5)	63 (50.4)
			χ^2^ = 9.24, df = 2, *p* = 0.010	χ^2^ = 6.97, df = 2, *p* = 0.031

° Eligible participants are all PLP surveyed (Total n: 1137); ^a^ Eligible participants who had not yet received at least one vaccination schedule or recall in the last 10 years for dTpa (Total n: 657); ^@^ Among PLP eligible for at least one cancer screening (Total n: 473); ^#^ Number of each item may not add up to total number of study population due to missing values.

**Table 3:** Uptake and willingness to be vaccinated in prison.

Vaccinations	Eligible	Vaccinations’ Uptake			Willingness to Be Vaccinated in Prison	
	N	Yes ^#^	Yes, in Prison	Yes, Out of Prison	Eligible	Yes
		N (%)	N (%) ^a^	N (%) ^a^	N (%)	N (%) ^a^
Catch-up and booster vaccination						
Diphtheria/tetanus/pertussis	1.137	1073 (94.4)	10 (0.9)	1.063 (93.5)	1.046 * (91.9)	432 (41.9)
Measles/mumps/rubella/varicella	1.137	43 (3.8)	16 (1.4)	27 (2.4)	1.094 (96.2)	280 (25.8)
Cancers-preventing vaccines						
Hepatitis B °	1.137	356 (31.3)	85 (7.5)	271 (23.8)	781 (68.7)	292 (38.2)
Papillomavirus	125	6 (4.8)	-	6 (4.8)	119 (95.2)	29 (24.4)
Vaccinations for individuals with specific conditions						
Meningococcal ACW135Y	154	3 (1.9)	1 (0.6)	2 (1.3)	151 (98.1)	61 (40.4)
Meningococcal B	154	2 (1.3)	1 (0.6)	1 (0.7)	152 (98.7)	63 (41.5)
Hepatitis A	70	2 (2.9)	2 (2.9)	-	68 (97.1)	26 (38.2)
Pneumococcal	251	7 (2.8)	2 (0.8)	5 (2)	244 (97.2)	82 (33.7)
Influenza	304	81 (26.7)	58 (19.1)	23 (7.6)	223 (73.4)	70 (31.4)
Herpes zoster	223	1 (0.4)	1 (0.4)	-	222 (100)	75 (34.4)

^#^ Having received vaccination during their lifetime; ° Recommended for all PLP; * Eligible are those who have never had a vaccination and/or recall in the last 10 years; ^a^ Number of each item may not add up to total number of study population due to missing value.

**Table 4:** Multilevel mixed-effects logistic regression models about having received (Model 1) and willingness to receive (Model 2) at least one vaccination schedule in prison according to several explanatory variables.

**Model 1. Having Received at Least One Vaccination Schedule in Prison**			
Log likelihood = −414.93, χ^2^ = 66.36 (16 df), *p* < 0.0001 No. of obs = 1.127			
Variable	OR	95% CI	*p*
Having at least one chronic disease			
No	1.00 *		
Yes	2.15	1.44–3.22	<0.001
Having received information regarding the recommended vaccination in prison			
No	1.00 *		
Yes	4.49	1.65–12.22	0.003
Length of detention in years, continuous	1.05	1.03–1.08	<0.001
Alcohol consumption (Audit-C)			
Being at risk of alcohol abuse	1.00 *		
Not being at risk of alcohol abuse	0.58	0.34–0.98	0.042
Never	0.81	0.52–1.27	0.366
Having at least one child			
No	1.00 *		
Yes	0.52	0.33–0.81	0.004
Gender			
Male	1.00 *		
Female	0.39	0.19–0.81	0.011
Age, continuous	0.99	0.97–1.01	0.149
Occupation before detention			
Unemployed	1.00 *		
Employed	0.71	0.47–1.08	0.112
Marital status			
Unmarried/widowed/separated/divorced	1.00 *		
Married/cohabitant	1.17	0.78–1.76	0.44
Nationality			
Foreigners	1.00 *		
Italians	1.19	0.61–2.33	0.603
Education level			
None/primary school/middle school	1.00 *		
High school/university degree	0.95	0.59–1.52	0.839
Sexual orientation			
Homosexual/bisexual	1.00 *		
Heterosexual	0.84	0.22–3.16	0.797
First Detention			
No	1.00 *		
Yes	1.41	0.91–2.18	0.131
Working activity in prison			
No	1.00 *		
Yes	0.82	0.55–1.24	0.351
Having participated in at least one smoking, alcohol or drug prevention program			
No	1.00 *		
Yes	1.09	0.71–1.69	0.668
**Model 2. Willingness to Receive at Least One Vaccination Schedule in Prison**			
Log likelihood = −403.16, χ^2^ = 47.78 (17 df), *p* < 0.0001 No. of obs = 653			
Variable	OR	95% CI	*p*
Nationality			
Foreigners	1.00 *		
Italians	2.05	1.09–3.86	0.026
Length of detention in years, continuous	1.03	1.01–1.06	0.023
Sexual orientation			
Homosexual/bisexual	1.00 *		
Heterosexual	0.06	0.01–0.56	0.013
Marital status			
Unmarried/widowed/separated/divorced	1.00 *		
Married/cohabitant	0.65	0.44–0.95	0.025
Alcohol consumption (Audit-C)			
Being at risk of alcohol abuse	1.00 *		
Not being at risk of alcohol abuse	0.61	0.36–1.01	0.050
Never	0.75	0.47–1.19	0.224
Age, continuous	0.98	0.96–1.01	0.132
Having at least one chronic disease			
No	1.00 *		
Yes	1.49	0.99–2.23	0.052
Occupation before detention			
Unemployed	1.00 *		
Employed	1.52	0.99–2.34	0.053
Having received information regarding the recommended vaccination in prison			
No	1.00 *		
Yes	1.64	0.37–7.35	0.514
Education level			
None/primary school/middle school	1.00 *		
High school/university degree	1.48	0.93–2.34	0.097
Having at least one child			
No	1.00 *		
Yes	1.19	0.74–1.92	0.478
Gender			
Male	1.00 *		
Female	1.11	0.53–2.34	0.782
First Detention			
No	1.00 *		
Yes	0.86	0.58–1.28	0.456
Working activity in prison			
No	1.00 *		
Yes	1.03	0.69–1.53	0.872
Having participated in at least one smoking, alcohol or drug prevention program			
No	1.00 *		
Yes	1.05	0.66–1.67	0.836
Lifetime drug use			
No	1.00 *		
Yes	1.42	0.95–2.12	0.085

* Reference category.

## Data Availability

The data that support the findings of this study are available from the corresponding author upon reasonable request, to ensure participants’ privacy and anonymity.

## Supplementary Materials

The following supporting information can be downloaded at: https://www.mdpi.com/article/10.3390/vaccines14070600/s1, Material S1: The independent variables included in the different final models, Material S2: Reasons reported by people living in prison for vaccination uptake (lifetime and during incarceration) and for willingness to uptake vaccination in prison.
