# Supplementary material for: Evaluating Vaccination Coverage and Willingness to Participate in Immunization Programs Among People Living in Prison: A Cross-Sectional Survey in Italy
Source: Vaccines (Basel). 2026 Jul 7;14(7):600. doi: 10.3390/vaccines14070600 (PMC13417343; doi:10.3390/vaccines14070600)
Supplement: Supplementary file 1 [file vaccines-14-00600-s001.zip › vaccines-4377054-supplementary.pdf]

Supplementary Material S1

The independent variables included in the different final models:

Model	Outcomes	Independent variables
1	**Having received at least one vaccination schedule in prison**<br>(No=0; Yes=1)	• age (continuous)°;<br>• gender (male=0; female=1)*;<br>• nationality (foreigners=0; Italians=1)°;<br>• sexual orientation (homosexual/bisexual=0; heterosexual=1)°;<br>• marital status (unmarried/widowed/separated/divorced=0; married/cohabitant=1)°;<br>• having at least one child (no=0; yes=1)*;<br>• education level (none/primary school/middle school=0; high school/university degree=1)°;<br>• occupation before detention (unemployed=0; employed=1)*;<br>• first detention (no=0; yes=1)°;<br>• length of detention, years (continuous)*;<br>• working activity in prison (no=0; yes=1)°;<br>• having participated in at least one preventing smoking, alcohol or drug program (no=0; yes=1)*;<br>• having at least one chronic disease (no=0; yes=1)*;<br>• having received information regarding the recommended vaccination in prison (no=0; yes=1)*;<br>• alcohol consumption (categorical) (never=1; not being at risk of alcohol abuse=1; being at risk of alcohol abuse=2)*.
2	**Willingness to receive at least one vaccinationschedule in prison**<br>(No=0; Yes=1)	• age (continuous)°;<br>• gender (male=0; female=1)°;<br>• nationality (foreigners=0; Italians=1)*;<br>• sexual orientation (homosexual/bisexual=0; heterosexual=1)*;<br>• marital status (unmarried/widowed/separated/divorced=0; married/cohabitant=1)*;<br>• having at least one child (no=0; yes=1)°;<br>• education level (none/primary school/middle school=0; high school/university degree=1)*;<br>• occupation before detention (unemployed=0; employed=1)°;

		• first detention (no=0; yes=1)°;<br>• length of detention, years (continuous)*;<br>• working activity in prison (no=0; yes=1)°;<br>• having participated in at least one preventing smoking, alcohol or drug program (no=0; yes=1)*;<br>• having at least one chronic disease (no=0; yes=1)*;<br>• having received information regarding the recommended vaccination in prison ((no=0; yes=1)°; alcohol consumption (categorical) (never=1; not being at risk of alcohol abuse=1; being at risk of alcohol abuse=2)*;<br>• lifetime drug use (no=0; yes=1)*.

*Included in the model if the bivariate test had a value of $p \leq 0.25$

°Included in the model if it was judged to potentially influence the investigated outcomes.

Supplementary material S2
Reasons reported by people living in prison for vaccination uptake (lifetime and during incarceration) and for willingness to uptake vaccination in prison°

			dTpa*	MMRV *	HBV*	HPV*	MenACWY*	MENB*	HAV*	PNEUMOCOCCAL*	INFLUENZA*	HERPES ZOSTER*
LIFETIME UPTAKE	REASON FOR NOT	Not mandatory	13 (2)	4 (0.4)	344 (45.8)	2 (1.7)	2 (1.3)	2 (1.3)	-	-	-	-
		Not perceived risk	11 (18.6)	-	48 (6.4)	3 (2.6)	12 (8)	9 (5.9)	1 (1.5)	15 (6.3)	27 (12.2)	17 (7.9)
		Availability was unknown	-	-	2 (0.3)	5 (4.4)	41 (27.2)	43 (28.3)	-	38 (16)	-	33 (15.3)
		Not considered useful	3 (5.1)	36 (3.3)	75 (10)	74 (64.4)	36 (23.8)	35 (23)	29 (43.3)	88 (37)	85 (38.5)	80 (37)
		Not offered in prison	2 (3.4)	33 (3)	76 (10.1)	1 (0.9)	23 (15.2)	-	14 (20.9)	35 (14.7)	46 (20.8)	31 (14.4)
		Not recommended by doctor	6 (10.2)	24 (2.2)	111 (14.8)	25 (21.7)	20 (12.7)	2 (13.8)	7 (10.5)	34 (14.3)	16 (7.2)	30 (13.9)
		Perceived lack of safety	3 (5.1)	1 (0.1)	12 (1.6)	-	7 (4.6)	7 (4.6)	1 (1.5)	3 (1.3)	13 (5.9)	9 (4.2)
		Perceived lack of efficacy	-	-	4 (0.5)	1 (0.9)	1 (0.7)	2 (1.3)	-	2 (0.8)	3 (1.4)	1 (0.5)
		Anti-vaccine stance	5 (8.5)	2 (0.2)	26 (3.5)	3 (2.6)	5 (3.3)	-	2 (3)	11 (4.6)	12 (5.4)	12 (5.6)
		Institutional distrust	-	-	8 (1.1)	1 (0.9)	2 (1.3)	1 (0.7)	-	1 (0.4)	2 (1)	2 (0.9)
		Access difficulties	8 (13.6)	1 (0.1)	12 (1.6)	2 (1.7)	4 (2.7)	4 (2.6)	-	6 (2.5)	10 (4.5)	4 (1.9)
		Fear	5 (8.5)	-	7 (0.9)	-	-	-	-	-	-	-
		Reason forgotten	3 (5.1)	5 (0.5)	30 (4)	-	-	-	7 (10.5)	-	-	-
		Unaware of the vaccination	1 (1.7)	-	-	-	-	-	-	-	-	33 (15.3)
		Past vaccine reactions	-	1 (0.1)	-	-	-	-	-	-	-	-
		High costs	-	-	-	-	-	-	1 (1.5)	-	-	-
		Previous infection	1 (1.7)	973 (89.5)	10 (1.3)	-	1 (0.7)	-	5 (7.5)	-	-	-
	REASON FOR YES	Mandatory	967 (92.8)	-	186 (53.1)	-	-	-	-	-	-	-
		Recommended by doctor	44 (4.2)	19 (46.3)	91 (26)	4 (50)	-	1 (50)	-	2 (28.6)	19 (23.75)	1 (33.3)
		Reduces infection risk	13 (1.3)	6 (14.6)	22 (6.3)	2 (25)	1 (33.3)	-	-	-	11 (13.75)	-
		Considered safe	7 (0.7)	1 (2.4)	6 (1.7)	-	1 (33.3)	1(50)	-	1 (14.3)	2 (2.5)	-
		Perceived efficacy	5 (0.5)	2 (4.9)	10 (2.9)	-	1 (33.3)	-	-	2 (28.6)	8 (10)	-
		Perceived risk of disease	7 (0.7)	2 (4.9)	10 (2.9)	-	1 (33.3)	-	-	3 (42.7)	22 (27.5)	2 (66.7)
		Offered in prison	1 (0.1)	-	11 (3.1)	-	1 (33.3)	-	-	-	26 (32.5)	-
		Pro-vaccination	9 (0.9)	4 (9.8)	5 (1.4)	-	-	-	-	1 (14.3)	-	-

**Institutional trust**		-	-	-	-	-	-	-	-	-
**Easy vaccine access**		-	1 (0.3)	-	-	-	-	-	-	-
**Free of charge**	2 (0.2)	-	-	-	-	-	-	-	-	-
**Prevention**	2 (0.2)	-	-	-	-	-	-	-	-	-
**Cut**	1 (0.1)	-	-	-	-	-	-	-	-	-
**Work requirement**	3 (0.3)	2 (4.9)	14 (4)	-	-	-	-	-	-	-
**Fear of contracting the disease**	1 (0.1)	-	-	-	-	-	-	-	-	-
**Parental decision**	7 (0.7)	4 (9.8)	-	-	-	-	-	-	-	-
**Previous infection**	-	1 (2.5)	-	-	-	-	-	-	-	1 (33.3)

			dTpa*	MMRV *	HBV*	HPV*	MenACWY*	MENB*	HAV*	PNEUMOCOCCAL*	INFLUENZA*	HERPES ZOSTER*
WILLINGNESS TO BE VACCINATED IN PRISON	REASON FOR NOT	**Not considered useful**	174(29)	343(45)	146 (31)	81 (90)	40 (44)	41 (46)	29 (69)	92 (58.6)	90 (59.21)	82 (57.75)
		**Perceived lack of safety**	89 (14.8)	59 (7.7)	65 (13.8)	-	10 (11)	10 (11.2)	3 (7.1)	16 (10.2)	17 (11.18)	15 (10.56)
		**Not effective**	37 (6.2)	25 (3.3)	19 (4)	2 (2.2)	4 (4.4)	4 (4.5)	1 (2.4)	9 (5.7)	3 (1.97)	8 (5.63)
		**Not perceived risk**	262 (43.7)	298 (39.1)	182 (38.6)	3 (3.3)	27 (29.7)	27 (30.3)	5 (11.9)	28 (17.8)	30 (19.74)	30 (21.13)
		**Advised against**	7 (1.2)	6 (0.8)	10 (2.1)	1 (1.1)	2 (2.2)	2 (2.3)	1 (2.4)	2 (1.3)	2 (1.32)	-
		**Anti-vaccine stance**	47 (7.8)	51 (6.7)	55 (11.7)	3 (3.3)	10 (11)	8 (9)	1 (2.4)	10 (6.4)	13 (8.55)	11 (7.75)
		**Institutional distrust**	25 (4.2)	24 (3.2)	23 (4.9)	2 (2.2)	2 (2.2)	2 (2.3)	1 (2.4)	3 (1.9)	6 (3.95)	3 (2.11)
		**High costs**	1 (0.2)	2 (0.3)	2 (0.4)	-	1(1.1)	1 (1.1)	-	1 (0.6)	1 (0.66)	1 (0.7)
		**Past vaccine reactions**	1 (0.2)	-	1 (0.2)	-	-	-	-	-	-	-
		**Fear**	8 (1.3)	1 (0.1)	-	-	2 (2.2)	2 (2.3)	-	2 (1.3)	1 (0.66)	1 (0.7)
		**Not recommended**	-	-	-	-	-	-	-	-	-	-
		**Access difficulties**	-	-	-	-	-	-	-	-	-	-
		**Other**	4 (0.7)	-	-	-	-	-	1 (2.4)	-	1 (0.66)	-
	REASON FOR YES	**Reduces infection risk**	138 (31.9)	109 (41.1)	102 (35.3)	15 (55.6)	24 (39.3)	26 (41.3)	9 (34.6)	30 (36.6)	24 (34.29)	28 (37.33)
		**Considered safe**	109 (25.2)	38 (14.3)	53 (18.3)	5 (18.5)	8 (13.1)	8 (12.7)	2 (7.7)	7 (8.5)	6 (8.57)	9 (12)
		**Perceived efficacy**	112 (25.9)	46 (17.4)	60 (20.8)	10 (37)	6 (9.8)	6 (9.5)	5 (19.2)	11 (13.4)	8 (11.43)	8 (10.67)
		**Perceived risk of disease**	99 (22.9)	45 (17)	73 (25.3)	4 (14.8)	20 (32.8)	22 (34.9)	7 (26.9)	30 (37.6)	25 (35.71)	26 (34.67)
		**Recommended by doctor**	9 (2.1)	8 (3)	18 (6.2)	2 (7.4)	6 (9.8)	6 (9.5)	-	4 (4.9)	5 (7.14)	5 (6.67)
		**Pro-vaccination**	39 (9)	39 (14.7)	34 (11.8)	-	1 (1.6)	1 (1.6)	5 (19.2)	2 (2.4)	5 (7.14)	3 (4)
		**Institutional trust**	2 (0.5)	1 (0.4)	2 (0.7)	-	-	-	-	-	-	-
		**It is free**	1 (0.2)	-	-	-	-	-	-	-	-	-
		**Mild side effects**	-	-	-	-	-	-	-	-	-	-
		**Outside prison option**	-	-	-	-	-	-	-	-	-	-
		**Legal obligation**	-	-	-	-	-	-	-	-	-	-
		**Caregiver role**	-	-	-	-	-	-	-	-	-	-
		**Other**	-	1 (0.4)	-	-	1 (1.6)	-	-	-	-	-

		dTpa*	MMRV *	HBV*	HPV*	MenACWY*	MENB*	HAV*	PNEUMOCOCCAL*	INFLUENZA*	HERPES ZOSTER*
UPTAKE IN PRISON	REASON FOR YES										
	**Mandatory**	2 (28.6)	1 (6.7)	35 (42.7)	-	-	-	-	-	-	-
	**Reduces infection risk**	-	3 (20)	15 (18.3)	-	-	-	-	-	10 (17.5)	-
	**Recommended by doctor**	1 (14.3)	7 (46.7)	17 (20.7)	-	1 (100)	1 (100)	-	1 (50)	6 (10.5)	-
	**Considered safe**	-	-	2 (2.4)	-	-	-	-	-	1 (1.8)	-
	**Perceived efficacy**	-	-	2 (2.4)	-	-	-	-	-	8 (14)	-
	**Perceived risk of disease**	1 (14.3)	1 (6.7)	4 (4.9)	-	-	-	-	1 (50)	13 (22.8)	1 (100)
	**Offered in prison**	1 (14.3)	-	11 (13.4)	-	-	-	-	-	25 (43.9)	-
	**Pro-vaccination**	2 (28.6)	2 (13.3)	-	-	-	-	-	-	-	-
	**Prevention**	1 (14.3)	-	-	-	-	-	-	-	-	-
	**Previous infection**	-	1 (6.7)	-	-	-	-	-	-	-	-
	**Other**	-	-	1 (1.2)	-	-	-	-	-	-	-

°Total percentages may exceed 100% as the questions allowed for multiple responses.

** N (%)*
